# Acanthocyte Sedimentation Rate as a Diagnostic Biomarker for Neuroacanthocytosis Syndromes: Experimental Evidence and Physical Justification

**DOI:** 10.3390/cells10040788

**Published:** 2021-04-02

**Authors:** Alexis Darras, Kevin Peikert, Antonia Rabe, François Yaya, Greta Simionato, Thomas John, Anil Kumar Dasanna, Semen Buvalyy, Jürgen Geisel, Andreas Hermann, Dmitry A. Fedosov, Adrian Danek, Christian Wagner, Lars Kaestner

**Affiliations:** 1 Experimental Physics, Saarland University, 66123 Saarbruecken, Germany; anra98@gmx.de (A.R.); francois.yaya@uni-saarland.de (F.Y.); grt.simionato@gmail.com (G.S.); thomas.john@physik.uni-saarland.de (T.J.); c.wagner@uni-saarland.de (C.W.); 2 Translational Neurodegeneration Section “Albrecht-Kossel”, Department of Neurology, University Medical Center Rostock, University of Rostock, 18051 Rostock, Germany; kevin.peikert@med.uni-rostock.de (K.P.); andreas.hermann@med.uni-rostock.de (A.H.); 3 Neurodegenerative Diseases, Department of Neurology, Technische Universität Dresden, 01062 Dresden, Germany; 4 Theoretical Medicine and Biosciences, Saarland University, 66424 Homburg, Germany; 5 Laboratoire Interdisciplinaire de Physique, UMR 5588, 38402 Saint Martin d’Hères, France; 6 Institute for Clinical and Experimental Surgery, Saarland University, 66424 Homburg, Germany; juergen.geisel@uks.eu; 7 Institute of Biological Information Processing and Institute for Advanced Simulation, Forschungszentrum Jülich, 52425 Jülich, Germany; a.dasanna@fz-juelich.de (A.K.D.); s.buvalyy@fz-juelich.de (S.B.); d.fedosov@fz-juelich.de (D.A.F.); 8 DZNE, German Center for Neurodegenerative Diseases, Research Site Rostock/Greifswald, 18051 Rostock, Germany; 9 Center for Transdisciplinary Neurosciences Rostock (CTNR), University Medical Center Rostock, University of Rostock, 18051 Rostock, Germany; 10 Neurologische Klinik und Poliklinik, Ludwig-Maximilians-Universität, 81366 Munich, Germany; adrian.danek@med.uni-muenchen.de; 11 Physics and Materials Science Research Unit, University of Luxembourg, 1511 Luxembourg, Luxembourg

**Keywords:** neuroacanthocytosis, erythrocyte sedimentation rate (ESR), diagnosis

## Abstract

(1) Background: Chorea-acanthocytosis and McLeod syndrome are the core diseases among the group of rare neurodegenerative disorders called neuroacanthocytosis syndromes (NASs). NAS patients have a variable number of irregularly spiky erythrocytes, so-called acanthocytes. Their detection is a crucial but error-prone parameter in the diagnosis of NASs, often leading to misdiagnoses. (2) Methods: We measured the standard Westergren erythrocyte sedimentation rate (ESR) of various blood samples from NAS patients and healthy controls. Furthermore, we manipulated the ESR by swapping the erythrocytes and plasma of different individuals, as well as replacing plasma with dextran. These measurements were complemented by clinical laboratory data and single-cell adhesion force measurements. Additionally, we followed theoretical modeling approaches. (3) Results: We show that the acanthocyte sedimentation rate (ASR) with a two-hour read-out is significantly prolonged in chorea-acanthocytosis and McLeod syndrome without overlap compared to the ESR of the controls. Mechanistically, through modern colloidal physics, we show that acanthocyte aggregation and plasma fibrinogen levels slow down the sedimentation. Moreover, the inverse of ASR correlates with the number of acanthocytes ($R^{2}=0.61$, p=0.004). (4) Conclusions: The ASR/ESR is a clear, robust and easily obtainable diagnostic marker. Independently of NASs, we also regard this study as a hallmark of the physical view of erythrocyte sedimentation by describing anticoagulated blood in stasis as a percolating gel, allowing the application of colloidal physics theory.

## 1. Introduction

Neuroacanthocytosis syndromes (NASs) are a group of rare genetic neurodegenerative diseases, the two core disorders being chorea-acanthocytosis (ChAc, OMIM #200150) and McLeod syndrome (MLS, OMIM #300842) [1,2,3,4,5,6,7]. While both diseases are characterized by neurological symptoms, such as hyper- and hypokinesia, epileptic seizures, and cognitive impairment, ChAc usually manifests much earlier in life (in the twenties) than MLS (usually after 40 years of age). The mean life expectancy after diagnosis has been reported to be approximately 11 years for ChAc and 21 years for MLS, leading to premature death in most cases [8]. The courses of the diseases are heterogeneous, usually slowly progressive, and often result in severe disability, while treatment options remain purely symptomatic so far [9]. Diagnosis is confirmed by genetic testing (VPS13A gene in ChAc, XK gene in MLS) and chorein expression detection by Western blot (ChAc) or immunohematological assessment (MLS) [5], although the initial detection often relies on blood smears [10]. Acanthocytes are deformed erythrocytes whose irregular membrane presents disordered, asymmetric spikes. The presence of acanthocytes among erythrocytes is one of the common features of NASs [4,5,6].

For these rare diseases, diagnosis is challenging, and misdiagnoses are frequent, which additionally increases the patients’ burden of disease. One of the reasons for delayed diagnosis is related to the difficult detection of acanthocytes in classical blood smears and routine laboratory testing: Sensitive detection requires special isotonically diluted blood samples [10], the number of acanthocytes varies among the patients, and their similarity to echinocytes often makes them hard to identify [6]. Therefore, it would be desirable to have an easier and more reliable initial diagnostic biomarker.

Here, we propose the erythrocyte sedimentation rate (ESR) as a convenient, inexpensive, and objective parameter that allows routine and systematic tests to be used to diagnose NASs. At the same time, we investigated the physical cause of the change in the acanthocyte sedimentation rate (ASR). In doing so, we devised a novel physical view of the sedimentation process based on colloidal physics that is in good agreement with the experimental data.

## 2. Materials and Methods

### 2.1. Patients and Blood Sampling

Blood samples were collected from six ChAc and three MLS patients. Patients were being treated at the neurologic departments of Technische Universität Dresden and the University Hospital of the Ludwig-Maximilian-Universität Munich. The diagnosis of ChAc and MLS was based on the clinical phenotype and was confirmed by detection of the VPS13A mutation and/or absence of the erythrocyte membrane protein chorein via Western blot (ChAc) or the detection of XK mutations (MLS) [11].

Blood sample collection was approved by the “Ärztekammer des Saarlandes”, ethics votum 51/18, and performed after informed consent was obtained according to the Declaration of Helsinki.

Blood was taken in the morning during routine patient visits and immediately transported to Saarland University in Homburg and Saarbrücken for further analysis, which started 6 to 8 h after withdrawal. Because transportation can have a tremendous effect on blood parameters [12], samples from relatives (if available) or the investigators were taken as transportation controls (in total, eight different individuals). This practice prevented us from matching patients and controls in terms of sex, age, or weight. Furthermore, three patients came directly to Saarland University, which allowed the investigation of fresh samples. However, concerning the ASR, there was no significant effect between fresh and transported samples, as outlined in Supplementary Figure S7. The sample collection and characterization were not blinded.

### 2.2. Measurements of the ESR

ESR/ASR measurements were performed in standard Westergren tubes (Dispette original, REF GS1500) with 200 mm height EthyleneDiamine Tetraacetic Acid (EDTA) blood. Color pictures of the tubes were automatically taken every minute for up to 50 h with a Canon EOS 500D camera to cover the whole range of sedimentation in every case. For each tube, a custom-written MATLAB algorithm extracted the position of the interface at the top of the concentrated erythrocyte suspension with an accuracy of at least 0.1 mm. Since the displacement of the interface between two successive frames was close to the picture resolution, instantaneous sedimentation velocities were extracted from least-squares fitting of the data points with cubic splines with 8 free interior knots, as allowed by the SLM (Shape Language Modeling) package [13].

### 2.3. Blood Smear Preparation and Evaluation

Blood smears were prepared as described by Storch et al. (2005) [10]. Whole EDTA blood was suspended in a 1:1 ratio in a 0.9% sodium chloride solution containing 5 IU/mL heparin and incubated for 1 h at room temperature. Then, a drop of this solution was smeared onto a glass coverslip by using a second coverslip. Dried smears were stained according to the Pappenheim method. First, they were placed in May–Grünwald solution for 3–4 min and then washed with distilled water. This was followed by staining with Giemsa solution for 20 min, followed by another washing step with distilled water. Images were visualized with an Olympus microscope (BX60, Olympus, Tokyo, Japan) with a 40× Plan Fluor Objective and 2× postmagnification, and they were recorded with a Charged Coupled Device camera (DP73, Olympus, Japan). At least 400 cells were counted by visual inspection and classified into stomatocytes, discocytes, echinocytes, acanthocytes, and “other” cell shapes.

### 2.4. Laboratory Parameters and Erythrocyte Preparation

When required, erythrocytes were washed by centrifugation (3000 rcf for 7 min). The supernatant was then removed and replaced by phosphate buffered saline (PBS) before redispensing the erythrocytes. This process was repeated three times. During the last iteration, the PBS was replaced by an adequate preparation (e.g., dextran diluted in PBS). Complete erythrocyte counts and plasma parameters were measured by standard methods in the Clinical Chemistry Laboratory of Saarland University Hospital (Homburg, Germany).

### 2.5. Widefield Microscopy

Microscopic pictures were taken using an inverted microscope in transmission mode with a bright field and a blue filter to enhance erythrocyte contrast with the background. A 20× objective was used to send pictures to a CCD camera. Samples were made by filling 30 μL well slides from Ibidi (Graefelfing, Germany, μ-Slide 18 Well, Catalog no 81826, inner diameter 5 mm, interior height 1.5 mm) with a hematocrit of 0.33%. The suspending liquid was either plasma from a control subject or patient or a preparation of dextran in PBS. The volume fraction of 0.33% was determined empirically to reach the percolation threshold, i.e., the smallest concentration that could produce percolating aggregates of erythrocytes on the lower side of the well when erythrocytes had sedimented. The final volume fraction at the bottom of the well after sedimentation was close to 45%. Pictures of an area of 610×480 μ$m^{2}$ of the sedimented layer were recorded and then analyzed through a custom-written MATLAB algorithm.

### 2.6. Two-Dimensional Simulations

In order to characterize the geometry and permeability of aggregates of various cell mixtures of healthy erythrocytes and acanthocytes, two-dimensional simulations were employed to study changes in the areas of the holes observed in such aggregates. These areas are the ones discussed in Section 3.3.1. Both healthy cells and acanthocytes were modeled as ring-like bead-spring chains, each consisting of 50 vertices, with a discocyte and star-like (having six rounded corners) shape, respectively. Even though the discocyte and star shapes had different areas, they had the same circumference, so that the aggregation interaction between cells did not depend on cell type. The total potential energy of the bead-spring chain model consists of three parts [14]. The first part is elastic energy, represented by nonlinear springs that connect neighboring vertices into a ring-like configuration. The second part accounts for the cellular bending resistance between two neighboring springs, with a bending modulus set to 50 k_B_T. Finally, the last contribution corresponds to an area constraint, which forces the discocyte shape of healthy erythrocytes with a fixed circumference. Note that acanthocytes were modeled as rigid bodies in the simulations, as they showed no significant deformations in previous experiments [15].

The simulations were performed in a domain of size 300×300 μ$m^{2}$ with periodic boundary conditions in both directions. Langevin dynamics with constant temperature were employed for time integration of the simulated system. Aggregation interactions between cells were modeled by the Lennard–Jones potential, $U\left(r\right)=4\epsilon ({\left(\sigma /r\right)}^{12}-{\left(\sigma /r\right)}^{6})$ for $r<r_{\mathrm{cut}}$ with σ=0.3μm and $r_{\mathrm{cut}}=0.72\;$μm. The strength of the potential, ϵ, was selected from the range of 1.5–2.5 k_B_T for different simulations to reflect aggregation variability in experiments. The total hematocrit ϕ was set to 50%. Two sets of simulations were performed, including one with only healthy erythrocytes and one with a mixture of 80% healthy cells and 20% acanthocytes (NAS case) by area fraction. Each set consisted of 11 simulations with different parameter values. Note that since the area of an acanthocyte is larger than that of a healthy cell, the total number of cells in the NAS case is smaller than that in the healthy case.

### 2.7. Measurement of Aggregation Forces

The aggregation forces between erythrocytes were measured through holographic optical tweezers as previously described [16]. This is a versatile technique for measuring forces in the piconewton (pN) range and allows force measurement at a single-cell level. A single infrared beam Nd:YAG laser (1064 nm, 3 W, Ventus 1064, Laser Quantum, Stockport, UK) was reflected by a parallel-aligned nematic liquid-crystal spatial light modulator (PAL-SLM, PPM X8267-15, Hamamatsu Photonics, Hamamatsu City, Shizuoka Pref., Japan) and focused with a large-numerical-aperture oil-immersion objective (60×, Nikon, Tokyo, Japan) in an inverted microscope (Nikon, TE 2000, Japan). The real-time manipulation of optical traps was made possible by imaging the sample with a Complementary Metal Oxide Semiconductor camera (ORCA Flash 4.0 V3, Hamamatsu, Japan) and using a MATLAB routine to compute the phase. The optical force holding cells could be tweaked by varying the initial laser power. Measurements were carried out according to the protocol depicted in Section 3.3.2. For each measurement, two erythrocytes with a discocytic shape were manually selected. Each erythrocyte was held by two optical traps placed at their extremities. The lower erythrocyte was brought into contact with the upper erythrocyte by changing the vertical position of the traps holding the erythrocyte in steps of 1 μm/s. The overlapping contact length between the erythrocytes was initially set to 4.5 μm for all the measurements. The aggregation force corresponds to the force that is insufficient to counterbalance the spontaneous aggregation forces between the two erythrocytes. The same protocol was repeated to measure at least 7 pairs of cells in both the pathological and healthy samples. Optical tweezers were calibrated, and forces were determined based on Stokes’ law, as in [17].

### 2.8. Statistical Analysis

Statistical analysis was performed in Prism8 (GraphPad Software Inc., San Diego, CA, USA). All datasets were checked for normality of the distribution with the Shapiro–Wilk test. The ESR for control, ChAc, and MLS patients was evaluated with the Brown–Forsythe and Welch analysis of variance (ANOVA) tests. Further significance between two conditions was then analyzed with an unpaired *t*-test, except if otherwise stated in the figure legend. The significance of *p*-values is abbreviated as n.s. (not significant) for *p* > 0.05, * for *p* < 0.05, ** for *p* < 0.01, *** for *p* < 0.001, and **** for *p* < 0.0001. Correlation analysis was performed by simple linear regression, and the *p*-value provides the slope difference from zero.

### 2.9. Confocal Microscopy and 3D Rendering

Approximately 5 μL of blood was placed into 1 mL of 0.1% glutaraldehyde (Sigma-Aldrich, St. Louis, MO, USA) solution in PBS in order to fix the shape of the red cells [18]. Erythrocytes (5 μL in 1 mL PBS) were stained with 5 μL of CellMaskTM Deep Red plasma membrane stain (0.5 mg/mL; Thermo Fisher Scientific, Waltham, MA, USA) for 24 h at room temperature. Then, the cells were washed 3 times by centrifugation at 4000 rcf for 5 min (Eppendorf Micro Centrifuge 5415 C, Brinkmann Instruments, Riverview, FL, USA) in 1 mL of PBS solution. After washing, the cells were resuspended in PBS and finally placed on a glass slide for confocal microscopy. Each labeled sample was placed between two glass slides for imaging (VWR rectangular coverglass, 24×60 ${\mathrm{mm}}^{2}$) by employing a piezo stepper for a 20 μm *z*-range. Confocal image generation was performed with a spinning disk-based confocal head (CSU-W1, Yokogawa Electric Corporation, Musashino, Japan). Image sequences were acquired with a digital camera (Orca-Flash 4.0, Hamamatsu Photonics, Japan). A custom-written MATLAB routine was used to crop single cells from each image and perform 3D reconstruction to enable visualization of the 3D shapes of the cells [19]. Each single-cell 3D image contained 68 individual planes with an extent of 100 by 100 pixels and a lateral (*x*/*y*) resolution of 0.11 μm/pixel. The piezo stepper had a minimal step width of 0.3 μm, defining the *z*-resolution. To compensate for the difference in resolution in the *x*/*y* and *z*-directions, we modified the *z*-scale by means of linear interpolation. Thus, the obtained *z*-stack had dimensions of 100×100×185 voxels. The image stacks were then passed to a custom-written ImageJ script. By applying a fixed threshold for every image, the script binarized the confocal *z*-stack to retrieve the cell membrane as an isosurface.

## 3. Results

### 3.1. Significance of the ESR in Neuroacanthocytosis Syndrome Patients

**Figure 1:**
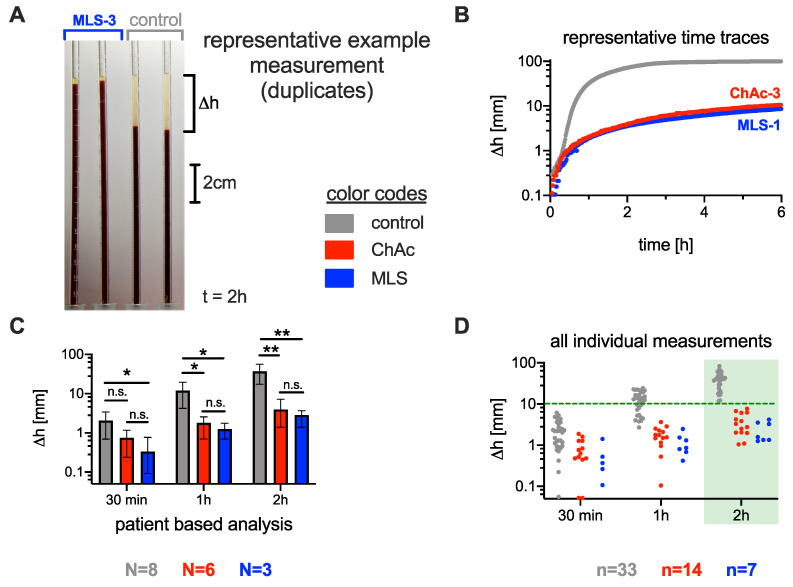
Comparison of the erythrocyte sedimentation rate (ESR) between neuroacanthocytosis patients and healthy controls. (**A**): ESR measurement setup: Standard Westergren tubes were filled with full blood and left to rest. The sedimentation height was measured over time. The picture was taken after 2 h. The first two tubes contain blood from an MLS patient, and the last two tubes contain blood from a healthy control donor. (**B**): Representative time traces of the sedimentation height for the different conditions. (**C**): Statistics on sedimentation height after typical times for healthy controls, chorea-acanthocytosis (ChAc) patients, and McLeod syndrome (MLS) patients. The bar height represents the average value, and the error bar depicts the range of individual values. Stars indicate the significance levels (Brown–Forsythe and Welch analysis of variance (ANOVA) test): n.s., not significant (*p*-value > 0.05); * *p* < 0.05; and ** *p* < 0.01. N refers to the number of patients. (**D**): Span of all individual sedimentation height measurements (each blood sample was measured at least in duplicate, and the blood of some patients was measured at different time points). After 2 h, a Δh of 10 mm could be regarded as a threshold to differentiate the ChAc and MLS conditions. The number n refers to the number of individual measurements out of the same number N of patients.

Time-resolved measurements of the ESR following the standard Westergren method were performed on six ChAc patients, three MLS patients, and eight healthy controls. The major hematological data, as well as the neurological phenotypes of the individuals, are given in Table 1. An image of the sedimentation tubes, the time curves of representative ASR/ESR measurements, the statistical presentation of patient-based data at selected time points, and the overview of all measurements at selected time points are summarized in Figure 1A–D, respectively.

Although the number of patients was limited, at 30 min, the difference in ESR was significantly lower for MLS patients than for controls (p=0.028). At 1 h, we found a significant difference between both ChAc patients (p=0.022) and MLS patients (p=0.017) and healthy controls. After 2 h, the *p*-value decreased to 0.006 for ChAc patients and to 0.005 for MLS patients relative to the healthy controls (Figure 1C).

However, a clear systematic difference between the whole range of measurements was only reached after 2 h. A sedimentation height of 10 mm can be regarded as a threshold (green dashed line in Figure 1D), i.e., only if the sedimentation phase is less than 10 mm within 2 h can the ESR indicate ChAc and MLS. These results do not show a significant sex dependence (cp. Supplementary Figure S1A).

As explained in the Methods, we prioritized having a transport control over matching other characteristics between patients and controls. Due to the circumstances of blood drawing, the controls were often either the mother of the patients or the researchers drawing the blood. The former were indeed older than the patients, while the latter were younger. From this perspective, this study can be considered as an initial orientation study, and we will improve this point in future research. However, for healthy groups, females tend to have higher ESR values, as do the elderly [20]. For male samples, our older patients had a lower ESR than the younger controls. Correcting the age bias would only increase the difference we observed. However, our limited sample size did not highlight a significant difference between the younger male controls and the older female controls. Since there is a significant difference when comparing the controls and the patients, the age or sex dependency is then likely lower than the influence of NASs.

### 3.2. Comparison of the Acanthocyte Percentage with the ESR

**Figure 2:**
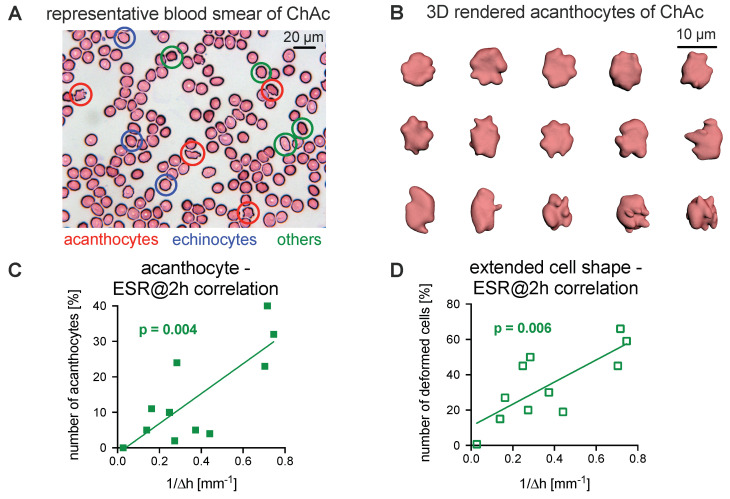
Relation of acanthocyte counts to ESR. (**A**): A representative stained blood smear of a ChAc patient. (**B**): Representative 3D-rendered acanthocytes from the freshly fixed blood of a ChAc patient. (**C**): Plot of the number of acanthocytes vs. the inverse of the ESR at 2 h and the corresponding correlation analysis. (**D**): Plot of the number of all deformed erythrocytes (echinocytes, acanthocytes, and other unspecified cell shapes) vs. the inverse of the ESR at 2 h and the corresponding correlation analysis. Panels C and D contain measurements from nine patients (cp. Table 1), where one patient was measured twice (independent blood sampling on different days). Since none of the controls presented with acanthocytes, they are summarized in one data point with their average ESR. The plotted line represents the linear regression. The analysis reveals a significant correlation for both parameters (panels C and D, $R^{2}$ coefficients of 0.61 and 0.59, respectively).

To date, the diagnosis of NASs has involved the detection of acanthocytes. Consequently, here, blood smears were prepared based on the protocol from Storch et al. [10] and later stained according to the Pappenheim method. An example is presented in Figure 2A. The manual count of cell shapes is prone to be subjective, biased, inaccurate, and, therefore, reproducible with only limited reliability, as outlined in Supplementary Figure S2. For a better assessment, we fixed fresh cells in 0.1% glutaraldehyde and performed confocal imaging of *z*-stacks to 3D render the erythrocytes. Figure 2B shows a number of representative acanthocytes in 3D, which were used for further analysis.

We observed that the ESR tends to decrease when the fraction of acanthocytes increases. To relate the ESR to the number of acanthocytes, the inverse of the ESR at 2 h was plotted as a function of the number of acanthocytes (Figure 2C). In a more general approach, we plotted the inverse of the ESR at 2 h against the number of all deformed cells (echinocytes, acanthocytes, and others; Figure 2D). The analysis revealed a significant correlation for both parameters ($R^{2}$ coefficient of 0.61 (acanthocytes) and 0.59 (all deformed cells), *p*-values of 0.004 and 0.006, respectively).

### 3.3. Explanations for Differences in the ESR

Because there was no significant difference in the ASR between ChAc and MLS patients (Figure 1), we did not discriminate between ChAc and MLS patients for further statistical comparisons. Therefore, we subsumed them into one NAS group.

The reasons for the differences in the ESR are varied and can depend on the erythrocytes, the blood plasma, or the erythrocyte–plasma volume relation (i.e., the hematocrit or erythrocyte volume fraction ϕ). The last reason can be excluded, since there were no significant differences in the hematocrit between the groups (cp. Table 1 and Supplementary Figure S1B). The primary physical parameters, such as the density of the individual erythrocytes represented by the mean cellular hemoglobin concentration (MCHC), as well as the constitution of the plasma, can also be part of the explanation. However, the MCHC indicated a higher density of erythrocytes in the NAS patients (Supplementary Figure S3C), a feature that would favor faster sedimentation; therefore, it was ruled out as an explanation. In contrast, the total plasma protein analysis (Supplementary Figure S4A), as a representative of the plasma content, indicated a lower protein concentration, and therefore, it served as a first hint for a plasma contribution to the ESR change. In the following sections, we present a more detailed view of the influence of both blood components—erythrocytes and plasma.

#### 3.3.1. Role of Erythrocytes in Sedimentation

The sedimentation of erythrocytes in blood plasma has been proposed, but was never established, to obey a so-called gel sedimentation regime [21,22]. Gel sedimentation is efficiently represented by several equations established for colloidal systems [23,24,25,26]. In particular, gel sedimentation can exhibit a delayed collapse [27,28]. Here, we apply the gel sedimentation model for sedimenting erythrocytes. This means that erythrocytes first experience only little sedimentation. Then, after a characteristic time on the order of a few minutes, some “cracks” or “channels” form within the aggregated structure of the erythrocytes, as suggested by a previous study [21]. We also observed this behavior in our samples (see Supplementary Figure S5). It is worthwhile to notice that, due to volume conservation, the sedimentation of the erythrocytes implies an upward flow of the plasma. The channel structures then enhance this upward flow of liquid. Here, we will apply, for the first time, some quantitative scaling based on these observations. Indeed, the maximal sedimentation speed $v_{M}$ following volume conservation and Darcy’s law [25] can be approximated as:

(1) $$v_{M}=\frac{k_{0}\Delta \rho g\phi_{0}}{\eta }\frac{2h_{c}}{\chi },$$

where $k_{0}$ is the permeability of the aggregated erythrocyte structure, Δρ is the density difference between the erythrocytes and the suspending liquid, $\phi_{0}$ is the initial hematocrit, η is the liquid viscosity, $h_{c}$ is the height of the channel, and χ is the characteristic radius of the zone where this channel influences the liquid flow [25]. Note that the permeability of the aggregated erythrocyte structure $k_{0}$ is proportional to the characteristic area of the holes within the cross-section 〈H〉 of aggregated erythrocytes: $k_{0}\propto \langle H\rangle$ [23]. Therefore, the maximal sedimentation speed is also proportional to 〈H〉:

(2) $$v_{M}\propto \langle H\rangle .$$

**Figure 3:**
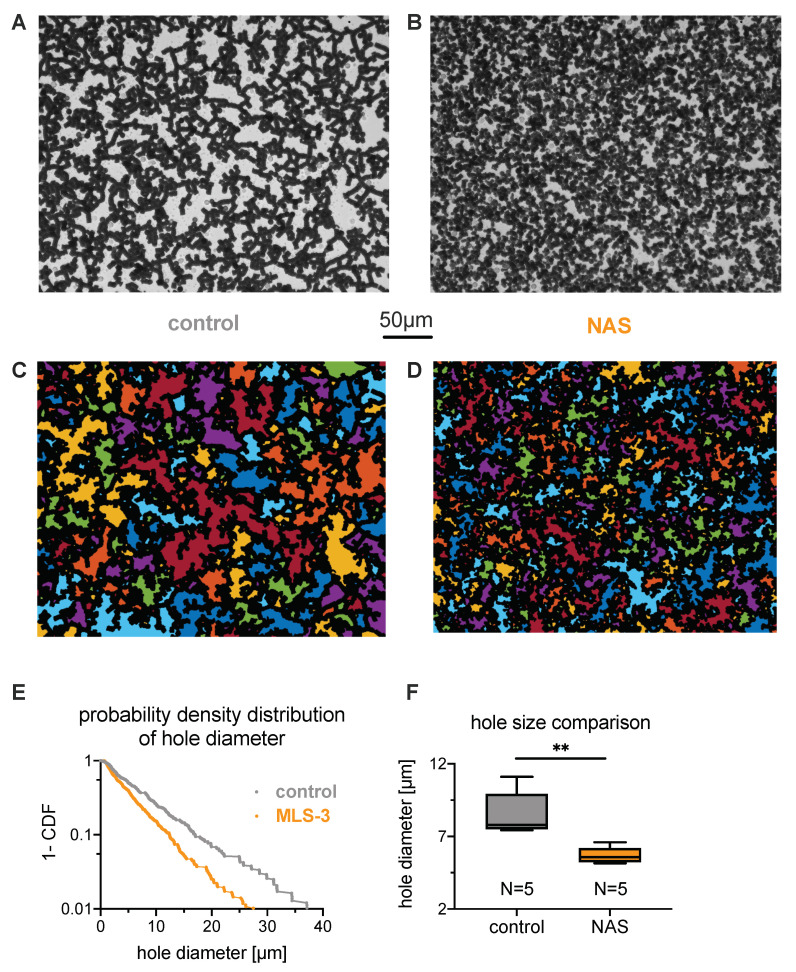

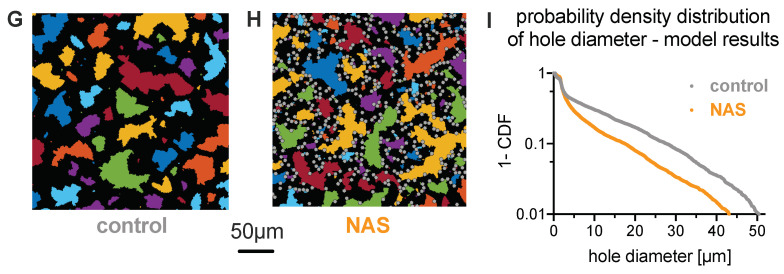
**Characteristics of sedimenting erythrocyte aggregates in autologous plasma.** (**A**,**B**): Sedimented layer of erythrocytes in plasma for a control subject and a patient on the bottom of a microscopy chamber after 2 h. Pictures were taken from the bottom of the well. (**A**): Healthy control donor and (**B**): patient (MLS-3). The volume fraction (hematocrit) in the microscope slide well (inner diameter 5 mm, height 1.5 mm) was initially 0.33% to reach a volume fraction close to 45% at the bottom of the well after sedimentation. (**C**,**D**): The same pictures as the previous, but with the holes detected in the percolating network of erythrocytes highlighted with random colors. The 50 μm scale is valid for panels (**A**) to (**D**). (**E**): Example of distributions of the square root of the hole areas, or hole diameter, shown as complementary cumulative density functions. The linear curve reflects an exponential distribution with the mean hole diameter as a single scale parameter. This scale parameter was estimated by the maximum likelihood method. (**F**): Statistical comparison of the scale parameters for control and neuroanthocytosis syndrome (NAS) erythrocytes in autologous plasma. The scale parameters are normally distributed among each population, and a *t*-test between the two populations indicates a significant difference (p=0.005, implying a significance level **, i.e. *p* < 0.01). (**G**): Simulation snapshot of healthy conditions with 100% discoid erythrocytes shown in black color, similarly to experimental images. (**H**): Simulated aggregate of a mixture of 80% normal erythrocytes (black area) and 20% acanthocytes (gray color) by area fraction. The scale bar is valid for panels (**G**,**H**). (**I**): Complementary cumulative density function of the square root of the hole areas from the simulations of healthy erythrocytes and the erythrocyte–acanthocyte mixture exemplified in (**G**,**H**).

To experimentally estimate the hole size, we allowed the erythrocytes to settle in a microscope chamber such that the final hematocrit on the coverslip layer was close to the physiological value of 45% (Figure 3A,B). This, when observing the chamber from the bottom, allowed a two-dimensional analysis of the hole size *H* based on microscopy images, as outlined in Figure 3C–E. The quantitative difference in average hole diameter (defined as the square root of the average hole area) is exemplified for one healthy control and one NAS patient by a probability density distribution (Figure 3E). A statistical analysis of the hole diameters is given in Figure 3F, revealing a larger hole size for the blood of healthy donors. One can also note that the total area covered by the erythrocytes is different between healthy controls and NAS patients (e.g., in Figure 3A–D, the total area of the holes is 8.5 $\times \;10^{4}\;$μ$m^{2}$ under control conditions, but 6.2 $\times \;10^{4}\;$μ$m^{2}$ for the patients). This was caused by the projection area of the cells despite the absolute hematocrit being equal between the groups. Under control conditions, almost all cells form rouleaux and, hence, contribute to a side projection, while acanthocytes and echinocytes have a larger projection area.

To better understand the effect of acanthocyte shape on the properties of sedimented aggregates (e.g., hole size), we performed a number of two-dimensional simulations with different compositions of healthy erythrocytes and acanthocytes; see the example snapshots in Figure 3G,H (and see Section 2.6 for simulation details). Figure 3I shows the corresponding cumulative density functions of hole diameter (defined as the square root of the hole area), which compare favorably to the experimental data in Figure 3E. The simulations reveal that acanthocyte shape and rigidity effectively reduce aggregation interactions, as they cannot maximize locally aggregated membrane areas between cells, which is achieved by cell deformation in healthy erythrocytes. A lower effective aggregation between cells leads to smaller hole sizes, which we confirmed directly by reducing the aggregation strength in the suspension of healthy cells.

**Figure 4:**
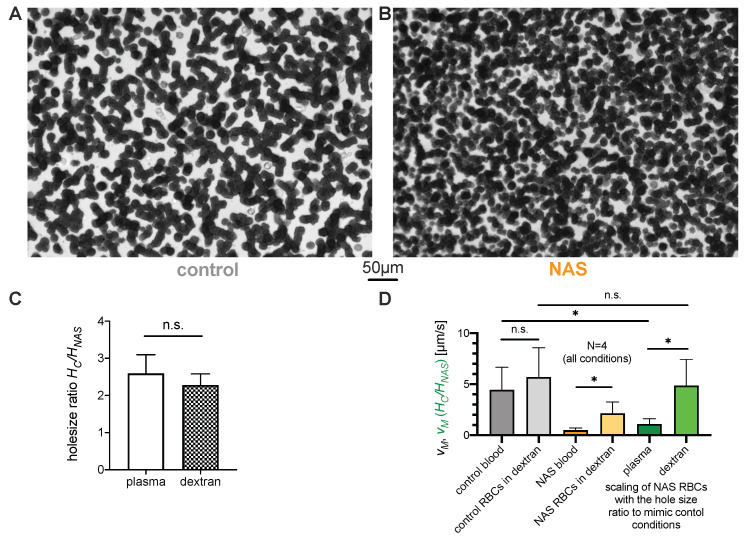
Characteristics of sedimenting erythrocyte aggregates in various suspension media. (**A**,**B**): Sedimented layer of erythrocytes in 55 mg/mL dextran from (**A**): a healthy control donor and (**B**): a NAS patient (MLS-3). The characteristic area of the holes in the network is visibly smaller in the network of the NAS patient’s erythrocytes. As a replacement medium, we used 70 kDa dextran diluted in PBS to a final mass concentration of 55 mg/mL. (**C**): The measured ratio of mean hole size for the suspension media (autologous plasma and dextran diluted in PBS with a mass concentration of 55 mg/mL). The dextran concentration was empirically chosen in order to achieve a sedimentation speed as close as possible to the rate obtained in the plasma of the healthy samples. (**D**): Characteristic (maximal) erythrocyte sedimentation velocities, with scaling based on aggregate geometries. The bars indicate the values of the characteristic erythrocyte sedimentation velocities. For each suspension medium, the patient scaling was obtained by multiplying the characteristic speed of each patient’s erythrocytes by the ratio of the characteristic hole areas, as justified by Equation (3). While the scaled velocity in autologous plasma is significantly smaller than the characteristic speed of the control erythrocytes, the scaling lies within the same range when the autologous plasma is replaced by a dextran-based medium. n.s., not significant (*p* > 0.05); * *p* < 0.05.

To further assess the role of erythrocytes in the reduced sedimentation rate and exclude the influence of individual plasma properties, sedimentation experiments with washed erythrocytes suspended in dextran (70 kDa) diluted in PBS were performed. A dextran concentration of 55 mg/mL, which mimics erythrocyte aggregation in blood samples of healthy controls, was empirically selected (Figure 4A,D). The aggregates formed in sedimented layers of erythrocytes in dextran solution (Figure 4A,B) indeed looked very similar to the aggregates in autologous plasma (compare with Figure 3A,B). The hole size ratio $H_{\mathrm{control}}/H_{\mathrm{NAS}}$ in the dextran solution was also very similar to that in the plasma solution (Figure 4C).

For further comparison, the maximum sedimentation speed $v_{M}$ was measured for each ESR test. Under the assumption of a fixed hematocrit of 45%, only the parameters $k_{0}$ and Δρ, which are related to the properties of erythrocytes, can imply a difference in sedimentation speed. Standard blood parameters, i.e., hemoglobin concentration (Table 1, Supplementary Figure S3A) and complete red blood cell count parameters, i.e., number of erythrocytes (Table 1, Supplementary Figure S3B) and mean cell volume (MCV; Supplementary Figure S3D), did not show any significant differences that could decrease the ESR of NAS patients through Δρ. However, MCHC (Supplementary Figure S3C) was slightly increased in the NAS patients, but, as previously mentioned, would then increase the ESR, and therefore, it cannot explain the differences. Thus, different cases could be compared by scaling the observed maximum sedimentation speed with the hole size; see Equation (2). If we denote patients with a subscript NAS and the healthy controls with C, then we obtain

(3) $$\frac{v_{C}}{v_{\mathrm{NAS}}}=\frac{\langle H_{C}\rangle }{\langle H_{\mathrm{NAS}}\rangle }\Rightarrow v_{C}=\frac{\langle H_{C}\rangle }{\langle H_{\mathrm{NAS}}\rangle }v_{\mathrm{NAS}}.$$

The $v_{M}$ of control ($v_{C}$) and NAS blood ($v_{NAS}$) as well as the corresponding erythrocyte–dextran mixtures are shown in Figure 4D (gray and orange columns, respectively). Additionally, the sedimentation speed was scaled according to Equation (3). This scaling is plotted in Figure 4D (green columns), along with the error bars corresponding to the standard deviation of the scaled speed measurements. The scaling makes the velocities comparable and indicates that the difference in the aggregate geometry as characterized by hole area accounts for the observed difference in sedimentation velocity when erythrocytes are suspended in a dextran-based medium. However, the scaling is not sufficient to explain the velocity ratio when the sedimentation is measured in autologous plasma, as shown in Figure 1. This indicates that the aggregate geometry accounts for some fraction of the ASR slowdown, while the remaining fraction must correspond to other contributions, such as patient plasma composition.

#### 3.3.2. Role of Plasma in the Sedimentation

**Figure 5:**
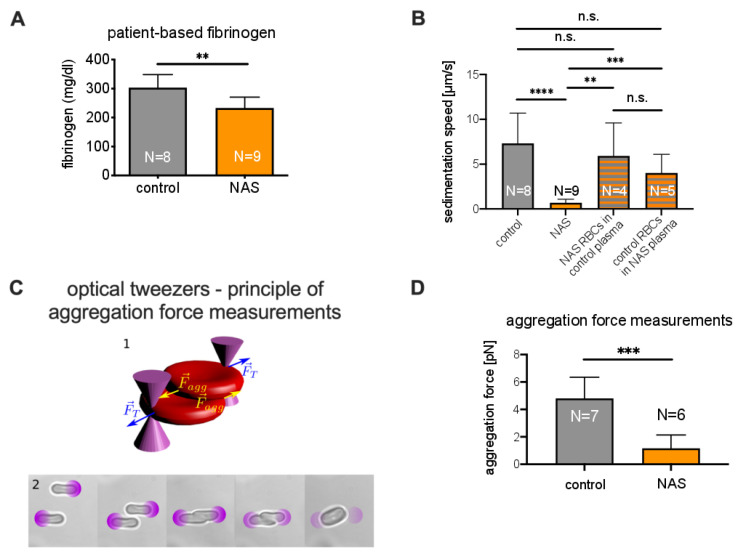
Measurements related to the role of plasma in sedimentation. (**A**): Comparison of fibrinogen concentrations. A significant difference consistent with the aggregation force difference was observed. (**B**): Comparison of sedimentation velocities of control samples, NAS patient samples, and samples with erythrocytes and plasma exchanged. (**C**) 1: Schematic of the forces acting on the erythrocytes trapped in optical tweezers. (**C**) 2: Microphotographs of the protocol for the measurement of the aggregating force. The purple circles show the locations of external optical traps. From left to right: After being selected, erythorcytes were lifted 15 μm from the microscope slide by four optical traps, one for each erythrocyte extremity. The optical traps had known trapping forces. The erythrocytes were then brought into contact. At equilibrium, the two inner traps were removed. The optical force holding the cells was decreased stepwise, and the overlap distance tended to increase in the same manner. Finally, spontaneous aggregation overcame the optical forces, and the erythrocytes escaped the trap. The trapping force at which the cells aggregated was considered to be the aggregation force. (**D**): Comparison of aggregation forces. A significant difference between patients and controls was observed. n.s., not significant (*p* > 0.05); ** *p* < 0.01; *** *p* < 0.001; and **** *p* < 0.0001.

As mentioned above, the total protein content likely contributes to the reduced ASR (Supplementary Figure S4A). Indeed, the concentration of plasma proteins, and particularly fibrinogen, is one of the first parameters ever described as influencing the ESR [29]. Therefore, we further analyzed plasma constituents, such as fibrinogen (Figure 5A), albumin (Supplementary Figure S4B), C-reactive protein (CRP, Supplementary Figure S4C), and immunoglobulin G (IgG, Supplementary Figure S4D). Since the last three did not show significant differences between NAS patients and healthy controls, we focused on fibrinogen, which was, on average, 23% lower in the patients (p=0.004).

To investigate the role of plasma in the increase in the ESR, we performed experiments in which the erythrocytes and plasma of patients were swapped with those of a healthy control with a matching blood group. We observed that healthy erythrocytes suspended in patient plasma sedimented significantly faster than NAS erythrocytes in healthy plasma (which confirms the importance of erythrocyte properties). However, patient erythrocytes also sedimented in healthy plasma significantly faster than in patient plasma, which is a clear sign that NAS plasma also slows sedimentation. Representative time traces are shown in Supplementary Figure S6. A statistical presentation is given in Figure 5B.

Previous reports have shown that an increase in fibrinogen concentration is related to an increased sedimentation speed and that fibrinogen has a larger impact on ESR than other proteins [30,31]. In particular, fibrinogen increases the interaction energy between erythrocytes [32]. To test whether this difference in interaction energy also occurs in NAS patients, we measured the aggregation forces between erythrocytes. To this end, we performed experiments using holographic optical tweezers, as shown in Figure 5C. As depicted in Figure 5D, the aggregation forces of erythrocytes from acanthocytosis patients in autologous plasma were significantly lower (p=0.0005) than those from healthy controls.

The details of the physical mechanisms leading to delayed collapse due to the appearance of cracks in weakly aggregated colloids are still under investigation [21,22,26,33,34,35,36]. However, recent studies highlighted that the aggregation energy can modify the sedimentation speed in this regime, as well as the time of onset [33,34,35,36]. Therefore, the difference in plasma fibrinogen concentration is likely to explain the further differences in sedimentation speed between NAS patients and healthy controls.

## 4. Discussion

We herein not only present a decreased ESR in NAS patients, but also conceptualize the biophysical reasons for this observation and highlight that measurement of the ESR needs to be implemented in routine clinical tests. Our measurements show that a decreased ESR can be used as an efficient biomarker to test for ChAc and MLS. For a sedimentation time of 2 h, we could (with our test tubes) determine a clear diagnostic threshold of 10 mm. In the context of a clinical workflow, the ESR (manually or automatically determined) is a robust and cost-efficient parameter that could easily complement the error-prone acanthocyte count. However, it is worthwhile to notice that some ChAc patients also do not present any acanthocytes (approximately 12% [37]). For these cases, further studies are needed to know if their ESR is still reduced. In any case, genetic analysis with molecular biology methods remains a necessary test for the identification of the type of NAS. Since the ESR also depends on the geometry of the tubes [20,38], systems other than those used here may need their own standardization.

Conventionally, ESR is used to detect general inflammation, which is indicated by an increased ESR [29,39]; however, there is no conflicting overlap because the change is in the opposite direction in NASs. We would have liked to test whether the ASR correlates to the severity of the disease, but there is no validated index or score for NAS severity available. Similarly to the acanthocyte count, the ASR cannot discriminate between ChAc and MLS. Furthermore, we cannot make a statement about other NASs (such as pantothenate kinase-associated neurodegeneration (PKAN)), but we suppose that a prolongation of the ESR also occurs in blood samples of patients with other diseases that show an increased number of acanthocytes.

Disease duration does not appear to be a good proxy for disease severity in this cohort, but probably also overall for NASs because of the heterogeneity of the phenotype. For instance, patient MLS-3 was very mildly affected, but already had a disease duration of 40 years (see Table 1) because he already had seizures when he was a teenager (which is likely to be related to MLS), but did not have any other symptoms for a long time. Additionally, no significant correlation between this duration and the acanthocyte number or the ESR was observed (Supplemental Figure S8).

The number of patients in our study may seem low (six ChAc and three MLS patients), but given the ultrararity of these diseases—the estimated prevalence is less than 1 to 5 per $10^{6}$ inhabitants each [5]—the number of participants can well be regarded as acceptable. In particular, this holds true because, with such a low number of patients, we were able to obtain significant results. Recent studies have identified promising drug targets, such as Lyn kinase, as a potential disease-modifying therapy for ChAc [5]. Considering the rarity and high variability of the natural history of the disease, there is an urgent need for a robust biomarker to achieve clinical trial readiness. The ASR might represent an ideal biomarker candidate in this context.

We have shown that both the erythrocyte properties and blood plasma compositions of NAS patients influence the ASR. Both components reduce the sedimentation speed, i.e., they are additive and provide a clear cut-off to differentiate from healthy individuals. This is, to some extent, surprising, since the ESR is a measure that integrates numerous parameters, and its initial intended use as a parameter for reporting inflammation has lost importance to a certain degree within the last decades [40] due to a lack of specificity.

Furthermore, we discussed physical mechanisms related to the erythrocyte and plasma properties that determine the ESR. Historical reports describe an altered aggregation behavior of acanthocytes [41] and the influence of erythrocyte shape on the ESR [42]. Here, we applied the principles of colloidal physics to describe sedimenting erythrocytes in a quantitative manner. Thus, we clearly show that the geometric properties of erythrocyte aggregates influence the porosity of sedimenting aggregates, which has a direct impact on the sedimentation velocity. Furthermore, a modeling approach provides evidence that the shapes of the erythrocytes (acanthocytes and echinocytes) and their rigidity are the primary cause for the differences in aggregate geometry. Regarding the plasma composition, we highlighted a difference in fibrinogen concentration, which modifies the interaction energy between erythrocytes. While the detailed influence of this energy on the sedimentation process is still under active discussion, it is clearly a key feature in the sedimentation process.

Future investigations are required for the clinical exploration of the ASR in a broader context of acanthocyte-related diseases, its longitudinal value in individual patients, and a deeper physical understanding of erythrocyte sedimentation. The clinical application comprises a broader test for neurodegenerative diseases, as well as the verification of the ASR as a biomarker for NAS treatments, as proposed above. For the mechanistic understanding of the process of erythrocyte sedimentation based on colloidal physics, we have provided an initial concept that also requires further research. These initial concepts could also be tested by comparing the ESR of other disorders that may show the presence of acanthocytes, e.g., hypolipoproteinemia [43], in further studies. Such a question would also provide some clinical interest and broaden the diagnostic application of the ESR.

## Figures and Tables

**Table 1:** Overview of the patients and controls.

Subjects	Sex	Age (y)	Hematocrit (%)	Hemoglobin (g/dL)	RBC Number ($10^{12}/L$)	Main Clinical Characteristics	Disease Duration	Acanthocyte Count (%)	Medication
ChAc-1	m	53	43	15.1	4.67	parkinsonism, dystonia, dysarthria, peripheral neuropathy, depression	15	40	scopoderm transdermal therapeutic system/day
ChAc-2	f	51	40	13.8	4.21	epilepsy, parkinsonism dystonia, dysarthria, peripheral neuropathy, cognitive impairment	30	5	levetiracetam 4 g/day, valproate 2 g/day, clobazam 10 mg/day, zonisamide 200 mg/day, vitamin D
ChAc-3	m	54	40	14.2	4.15	epilepsy, parkinsonism, dystonia, dysarthria, dysphagia, peripheral neuropathy, cognitive impairment	21	10	lamotrigine 110 mg/day, oxcarbazepine 1.5 g/day, lacosamide 300 mg/day, levodopa 300 mg/day, esomeprazole 40 mg/day
ChAc-4	m	34	46	16.2	5.40	drug-resistant epilepsy, mild chorea, tics, cognitive impairment, peripheral neuropathy, myopathy	11	4	lacosamide 550 mg/day, zonisamide 300 mg/day, perampanel 4 mg/day, vitamin D, PRN: lorazepam/midazolam
ChAc-5	m	29	43	15.7	4.91	drug-resistant epilepsy, mild chorea, tics, cognitive impairment, irritability, anxiety, depression, psychosis	15	11	lacosamide 600 mg/day, zonisamide 400 mg/day, mirtazapine 15 mg/day, olanzapine 2.5 mg/day, vitamin D, PRN: lorazepam/midazolam
ChAc-6	m	42	41	15.2	4.96	epilepsy, feeding dystonia, orofacial dyskinesia, chorea, peripheral neuropathy, myopathy, impulse control disorder	12	5	levetiracetame 1 g/day, quetiapine 400 mg/day, ramipril 2.5 mg/day, metoprolol 47.5 mg/day, PRN: metamizole, ibuprofen, pantoprazole
MLS-1	m	58	43	16.0	4.82	myopathy	15	2	magnesium, St. John’s wort extract, pumpkin seed preparation, PRN: pantoprazol
MLS-2	m	51	42	15.3	4.67	epilepsy, peripheral neuropathy, myopathy	40	32	levetiracetame 2 g/day, lamotrigine 400 mg/day, candesartan 8 mg/day, vitamin D, PRN: methylprednisolone
MLS-3	m	52	45	16.2	5.17	neuropathy, myopathy	18	24	No medication
female controls (average, N = 4)		64 ± 10	38.5 ± 2.6	12.6 ± 1.3	4.36 ± 0.19	—	—	0	—
male controls (average, N = 4)		38 ± 11	43.4 ± 2.1	15.5 ± 0.8	5.17 ± 0.1	—	—	0	—

## Data Availability

The data presented in this study are available in the main Figures and the Supplementary Material. Intermediate measurements can be obtained from corresponding authors on reasonable request.

## Supplementary Materials

The following are available online at https://www.mdpi.com/article/10.3390/cells10040788/s1, Figure S1: Sex-specific comparisons; Figure S2: Analysis of blood smears; Figure S3: Comparison of red blood cell properties; Figure S4: Comparison of plasma parameters; Figure S5: Channels that appear in sedimenting blood; Figure S6: Example of time traces obtained by switching control and patient plasma; Figure S7: Comparison of fresh and delayed observations; Figure S8: Correlation between disease duration and the diagnostic variables.

## Abbreviations

The following abbreviations are used in this manuscript:

ESR	Erythrocyte sedimentation rate
ASR	Acanthocyte sedimentation rate
NAS	Neuroacanthocytosis syndrome
MLS	McLeod syndrome
ChAc	Chorea-acanthocytosis
PRN	Pro Re Nata: administration timing of prescribed medication is left to the patient/caregiver
PBS	Phosphate buffered saline
EDTA	EthyleneDiamine Tetraacetic Acid

## References

[1] Adam M.P., Ardinger H.H., Pagon R.A., Wallace S.E., Bean L.J., Stephens K., Amemiya A. (1993). McLeod neurocanthocytosis Syndrom. GeneReviews^®^.

[2] Danek A., Rubio J.P., Rampoldi L., Ho M., Dobson-Stone C., Tison F., Symmans W.A., Oechsner M., Kalckreuth W., Watt J.M. (2001). McLeod neuroacanthocytosis: Genotype and phenotype. Ann. Neurol..

[3] Baeza A.V., Dobson-Stone C., Rampoldi L., Bader B., Walker R.H., Danek A., Monaco A.P. (2019). Chorea-acanthocytosis. GeneReviews^®^.

[4] Siegl C., Hamminger P., Jank H., Ahting U., Bader B., Danek A., Gregory A., Hartig M., Hayflick S., Hermann A. (2013). Alterations of red cell membrane properties in nneuroacanthocytosis. PLoS ONE.

[5] Peikert K., Danek A., Hermann A. (2018). Current state of knowledge in Chorea-Acanthocytosis as core Neuroacanthocytosis syndrome. Eur. J. Med. Genet..

[6] Adjobo-Hermans M.J., Cluitmans J.C., Bosman G.J. (2015). Neuroacanthocytosis: Observations, theories and perspectives on the origin and significance of acanthocytes. Tremor Other Hyperkinetic Mov..

[7] Rubio J.P., Danek A., Stone C., Chalmers R., Wood N., Verellen C., Ferrer X., Malandrini A., Fabrizi G.M., Manfredi M. (1997). Chorea-acanthocytosis: Genetic linkage to chromosome 9q21. Am. J. Hum. Genet..

[8] Walker R.H., Miranda M., Jung H.H., Danek A. (2019). Life expectancy and mortality in chorea-acanthocytosis and McLeod syndrome. Parkinsonism Relat. Disord..

[9] Walker R.H. (2015). Management of neuroacanthocytosis syndromes. Tremor Other Hyperkinetic Mov..

[10] Storch A., Kornhass M., Schwarz J. (2005). Testing for acanthocytosis. J. Neurol..

[11] Dobson-Stone C., Velayos-Baeza A., Filippone L.A., Westbury S., Storch A., Erdmann T., Wroe S.J., Leenders K.L., Lang A.E., Dotti M.T. (2004). Chorein detection for the diagnosis of chorea-acanthocytosis. Ann. Neurol..

[12] Makhro A., Huisjes R., Verhagen L.P., Manu-Pereira M.d.M., Llaudet-Planas E., Petkova-Kirova P., Wang J., Eichler H., Bogdanova A., van Wijk R. (2016). Red cell properties after different modes of blood transportation. Front. Physiol..

[13] D’Errico J. (2009). SLM-Shape Language Modeling. Mathworks. http://www.mathworks.com/matlabcentral/fileexchange/24443-slm-shape-language-modeling.

[14] Zhang Z., Henry E., Gompper G., Fedosov D.A. (2015). Behavior of rigid and deformable particles in deterministic lateral displacement devices with different post shapes. J. Chem. Phys..

[15] Cluitmans J.C., Tomelleri C., Yapici Z., Dinkla S., Bovee-Geurts P., Chokkalingam V., De Franceschi L., Brock R., Bosman G.J. (2015). Abnormal red cell structure and function in neuroacanthocytosis. PLoS ONE.

[16] Steffen P., Jung A., Nguyen D.B., Müller T., Bernhardt I., Kaestner L., Wagner C. (2011). Stimulation of human red blood cells leads to Ca2+-mediated intercellular adhesion. Cell Calcium.

[17] Svoboda K., Block S.M. (1994). Biological applications of optical forces. Annu. Rev. Biophys. Biomol. Struct..

[18] Abay A., Simionato G., Chachanidze R., Bogdanova A., Hertz L., Bianchi P., Van Den Akker E., Von Lindern M., Leonetti M., Minetti G. (2019). Glutaraldehyde—A subtle tool in the investigation of healthy and pathologic red blood cells. Front. Physiol..

[19] Simionato G., Hinkelmann K., Chachanidze R., Bianchi P., Fermo E., van Wijk R., Leonetti M., Wagner C., Kaestner L. (2021). Red blood cell phenotyping from 3D confocal images using artificial neural networks. PLoS Comput. Biol..

[20] Brigden M.L., Page N.E. (1993). Three closed-tube methods for determining erythrocyte sedimentation rate. Lab. Med..

[21] Pribush A., Meyerstein D., Meyerstein N. (2010). The mechanism of erythrocyte sedimentation. Part 1: Channeling in sedimenting blood. Colloids Surf. B Biointerfaces.

[22] Pribush A., Meyerstein D., Meyerstein N. (2010). The mechanism of erythrocyte sedimentation. Part 2: The global collapse of settling erythrocyte network. Colloids Surf. B Biointerfaces.

[23] Allain C., Cloitre M., Wafra M. (1995). Aggregation and sedimentation in colloidal suspensions. Phys. Rev. Lett..

[24] Manley S., Skotheim J., Mahadevan L., Weitz D.A. (2005). Gravitational collapse of colloidal gels. Phys. Rev. Lett..

[25] Derec C., Senis D., Talini L., Allain C. (2003). Rapid settling of a colloidal gel. Phys. Rev. E.

[26] Senis D., Gorre-Talini L., Allain C. (2001). Systematic study of the settling kinetics in an aggregating colloidal suspension. Eur. Phys. J. E Soft Matter.

[27] Buscall R., Choudhury T.H., Faers M.A., Goodwin J.W., Luckham P.A., Partridge S.J. (2009). Towards rationalising collapse times for the delayed sedimentation of weakly-aggregated colloidal gels. Soft Matter.

[28] Lindström S.B., Kodger T.E., Sprakel J., Weitz D.A. (2012). Structures, stresses, and fluctuations in the delayed failure of colloidal gels. Soft Matter.

[29] Bedell S.E., Bush B.T. (1985). Erythrocyte sedimentation rate. From folklore to facts. Am. J. Med..

[30] Gray S.J., Mitchell E.B., Dick G. (1942). Effect of purified protein fractions on sedimentation rate of erythrocytes. Proc. Soc. Exp. Biol. Med..

[31] Flormann D., Kuder E., Lipp P., Wagner C., Kaestner L. (2015). Is there a role of C-reactive protein in red blood cell aggregation?. Int. J. Lab. Hematol..

[32] Brust M., Aouane O., Thiébaud M., Flormann D., Verdier C., Kaestner L., Laschke M., Selmi H., Benyoussef A., Podgorski T. (2014). The plasma protein fibrinogen stabilizes clusters of red blood cells in microcapillary flows. Sci. Rep..

[33] Ghazali M.E.B., Argo Y., Kyotoh H., Adachi Y. (2020). Effect of the concentration of NaCl and cylinder height on the sedimentation of flocculated suspension of Na-montmorillonite in the semi-dilute regime. Paddy Water Environ..

[34] Padmanabhan P., Zia R. (2018). Gravitational collapse of colloidal gels: Non-equilibrium phase separation driven by osmotic pressure. Soft Matter.

[35] Razali A., Fullerton C.J., Turci F., Hallett J.E., Jack R.L., Royall C.P. (2017). Effects of vertical confinement on gelation and sedimentation of colloids. Soft Matter.

[36] Harich R., Blythe T., Hermes M., Zaccarelli E., Sederman A., Gladden L.F., Poon W.C. (2016). Gravitational collapse of depletion-induced colloidal gels. Soft Matter.

[37] Rampoldi L., Danek A., Monaco A.P. (2002). Clinical features and molecular bases of neuroacanthocytosis. J. Mol. Med..

[38] Patton W., Meyer P., Stuart J. (1989). Evaluation of sealed vacuum extraction method (Seditainer) for measurement of erythrocyte sedimentation rate. J. Clin. Pathol..

[39] Kratz A., Plebani M., Peng M., Lee Y., McCafferty R., Machin S., International Council for Standardization in Haematology (ICSH) (2017). ICSH recommendations for modified and alternate methods measuring the erythrocyte sedimentation rate. Int. J. Lab Hematol..

[40] Brigden M.L. (1999). Clinical utility of the erythrocyte sedimentation rate. Am. Fam. Phys..

[41] Salt H., Wolff O., Lloyd J., Fosbrooke A., Cameron A., Hubble D. (1960). On having no beta-lipoprotein. A syndrome comprising *α*-beta-lipoproteinaemia, acanthocytosis, andsteatorrhoea. Lancet.

[42] Reinhart W.H., Singh A., Straub P.W. (1989). Red blood cell aggregation and sedimentation: The role of the cell shape. Br. J. Haematol..

[43] Brecher G., Bessis M. (1972). Present status of spiculed red cells and their relationship to the discocyte-echinocyte transformation: A critical review. Blood.

