# Supplementary material for: Acanthocyte Sedimentation Rate as a Diagnostic Biomarker for Neuroacanthocytosis Syndromes: Experimental Evidence and Physical Justification
Source: Cells. 2021 Apr 2;10(4):788. doi: 10.3390/cells10040788 (PMC8067274; doi:10.3390/cells10040788)
Supplement: Supplementary file 1 [file cells-10-00788-s001.pdf]

Supplementary Materials: Acanthocyte sedimentation rate as a diagnostic biomarker for neuroacanthocytosis syndromes: experimental evidence and physical justification

Alexis Darras [1,†*], Kevin Peikert [2,3,†], Antonia Rabe [1,4], François Yaya [1,5], Greta Simionato [1,6], Thomas John[1], Anil Kumar Dasanna [7], Semen Buvalyy [7], Jürgen Geisel[8], Andreas Hermann[2,3,9,10], Dmitry A. Fedosov[7], Adrian Danek[11], Christian Wagner[1,12] and Lars Kaestner [1,4*]

1. Supplementary Figures

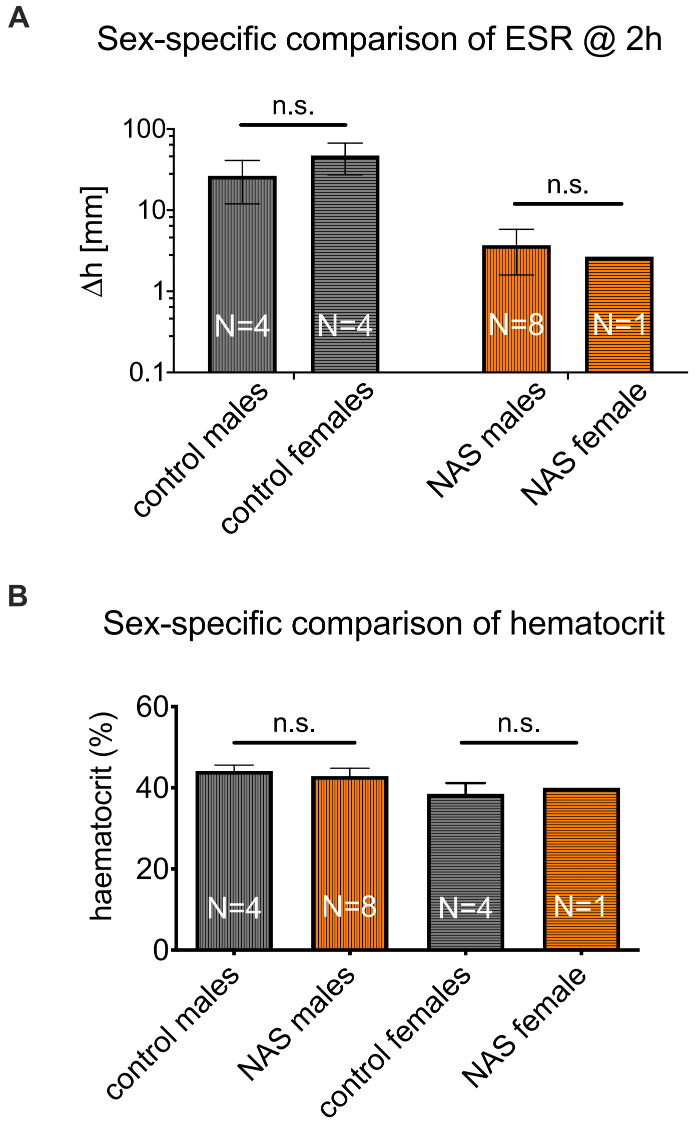

Supplementary Figure S1. Sex-specific comparisons. A: Sex-specific comparison of the ESR after 2 h. In the control group, males and females are equally distributed, while in the patient group, the higher prevalence of the disease in males is reflected by the presence of only one female ChAc patient. We did not detect any significant sex-based difference between the controls and patients. B: Comparison of hematocrit. Since gender differences in the hematocrit are known, we compared this parameter separately for males and females between control and NAS blood samples. We did not find significant differences between healthy controls and NAS patients either in the gender-specific comparison as outlined here or when male and female subjects were pooled.

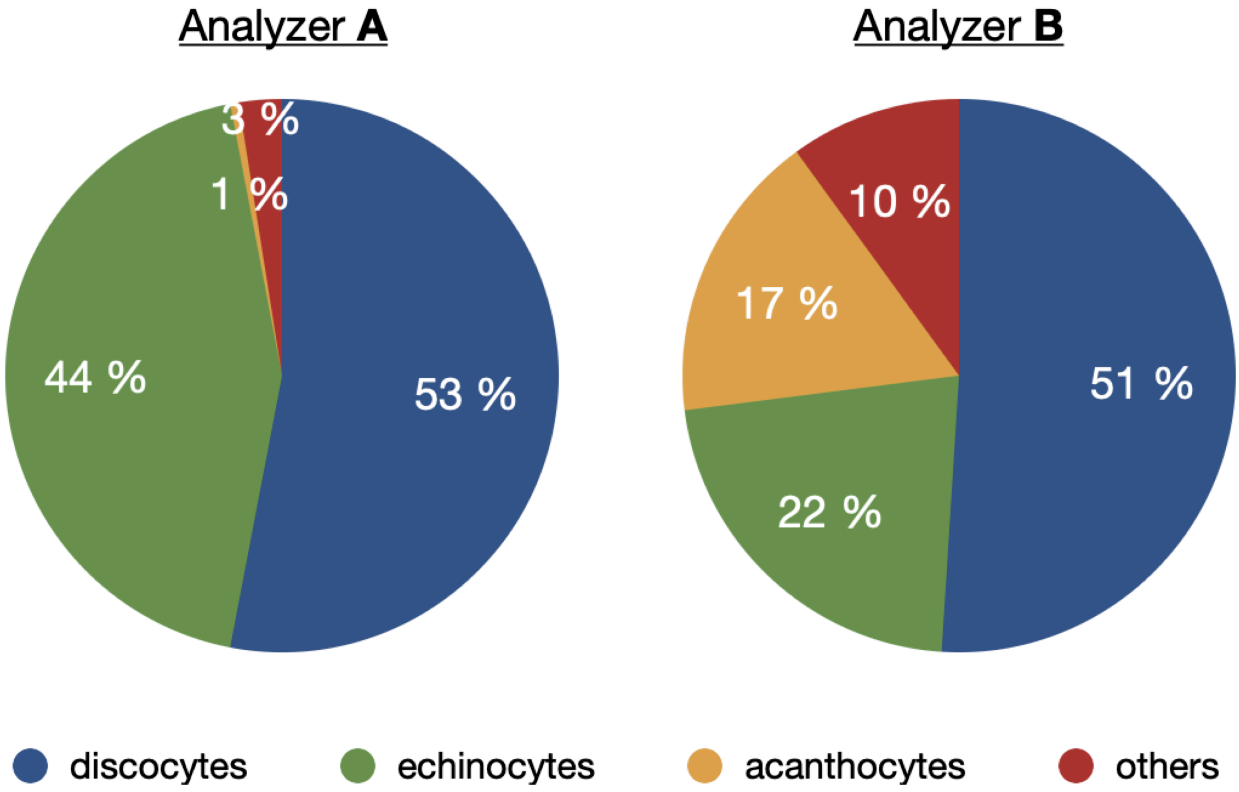

Supplementary Figure S2. Analysis of blood smears. The blood smear from patient ChAc-5 was analyzed by two skilled staff members familiar with erythrocyte shapes. They were blinded to the intended comparison when they performed the counts. Approximately 1000 erythrocytes were counted by each analyzer. While the number of discocytes was consistent, the number of echinocytes, acanthocytes and other erythrocyte shapes (including stomatocytes and other cell shapes of known or unknown categories) varied tremendously. These differences reflect the uncertainty of acanthocyte counting based on blood smears.

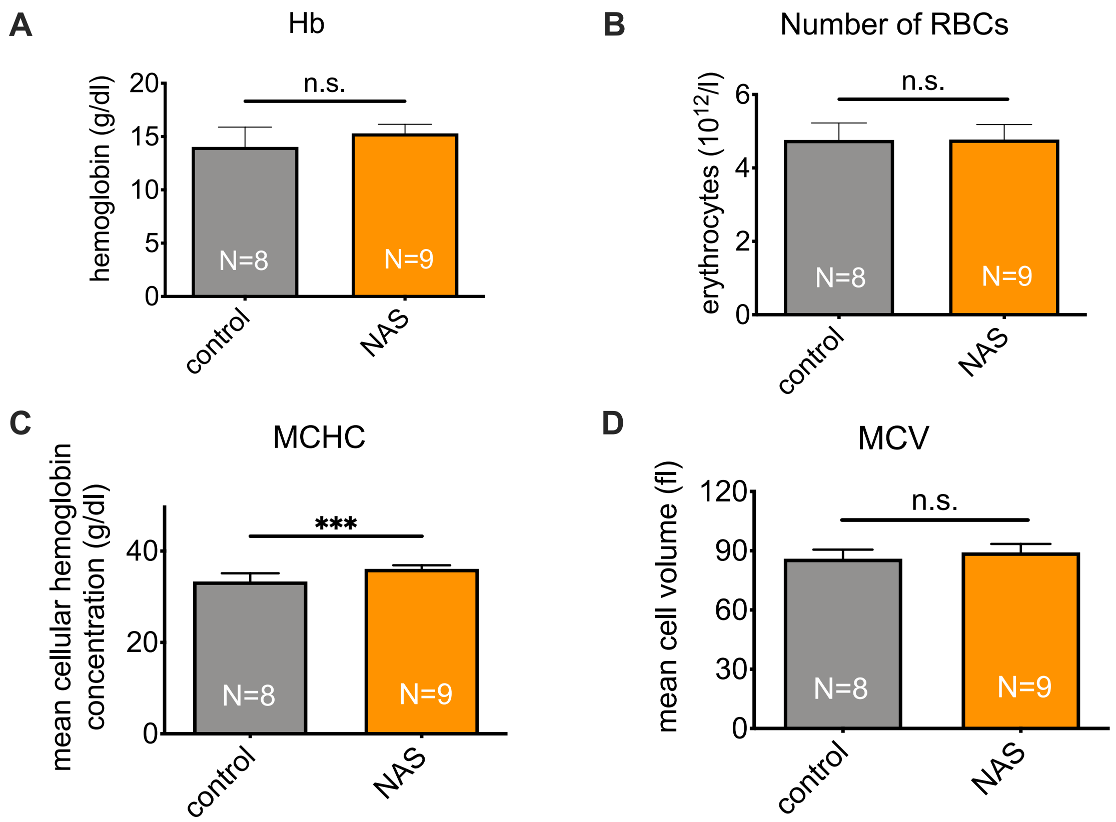

Supplementary Figure S3. Comparison of red blood cells properties. A: Comparison of the hemoglobin concentration. For the hemoglobin (Hb) concentration, there was no significant difference between healthy controls and NAS patients. B: Comparison of the number of erythrocytes. For the number of erythrocytes (RBCs) per volume, there was no significant difference between healthy controls and NAS patients. C: Comparison of the mean cellular hemoglobin concentration. The mean cellular hemoglobin concentration (MCHC) was significantly increased in NAS patients. This is surprising since there were no differences in hematocrit (Supplemental Figure S1B), hemoglobin (panel A), number of erythrocytes (panel B) or mean cellular volume (panel D). The difference cannot be explained by the unequal gender distribution in the two groups. D: Comparison of the mean erythrocyte volume. For the mean cellular volume (MCV) of erythrocytes, there was no significant difference between healthy controls and NAS patients.

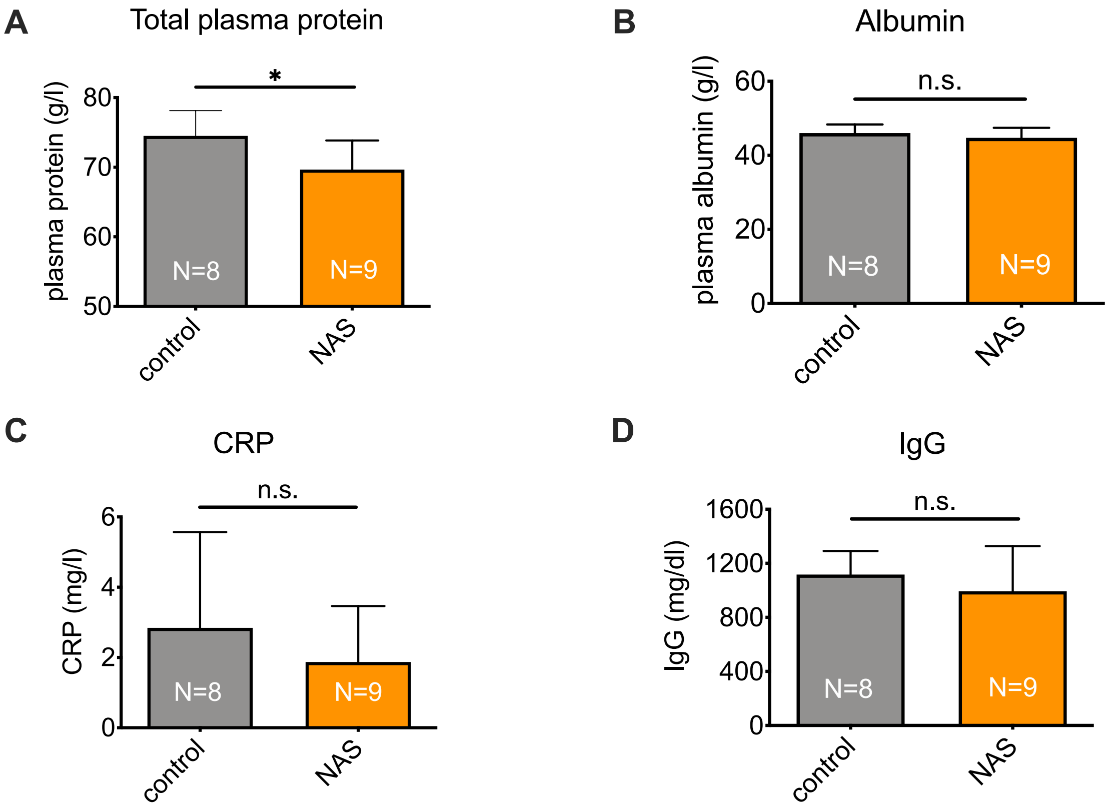

Supplementary Figure S4. Comparison of plasma parameters. A: Comparison of total plasma protein. The total plasma protein was reduced in NAS patients ($p = 0.023$), providing an initial hint for the contribution of blood plasma to the reduced ESR. B: Comparison of albumin concentration. For the albumin concentration, there was no significant difference between healthy controls and NAS patients. C: Comparison of the C-reactive protein concentration. For the C-reactive protein (CRP) concentration, there was no significant difference between healthy controls and NAS patients. D: Comparison of the immunoglobulin G concentration. For the immunoglobulin G (IgG) concentration, there is no significant difference between healthy controls and NAS patients.

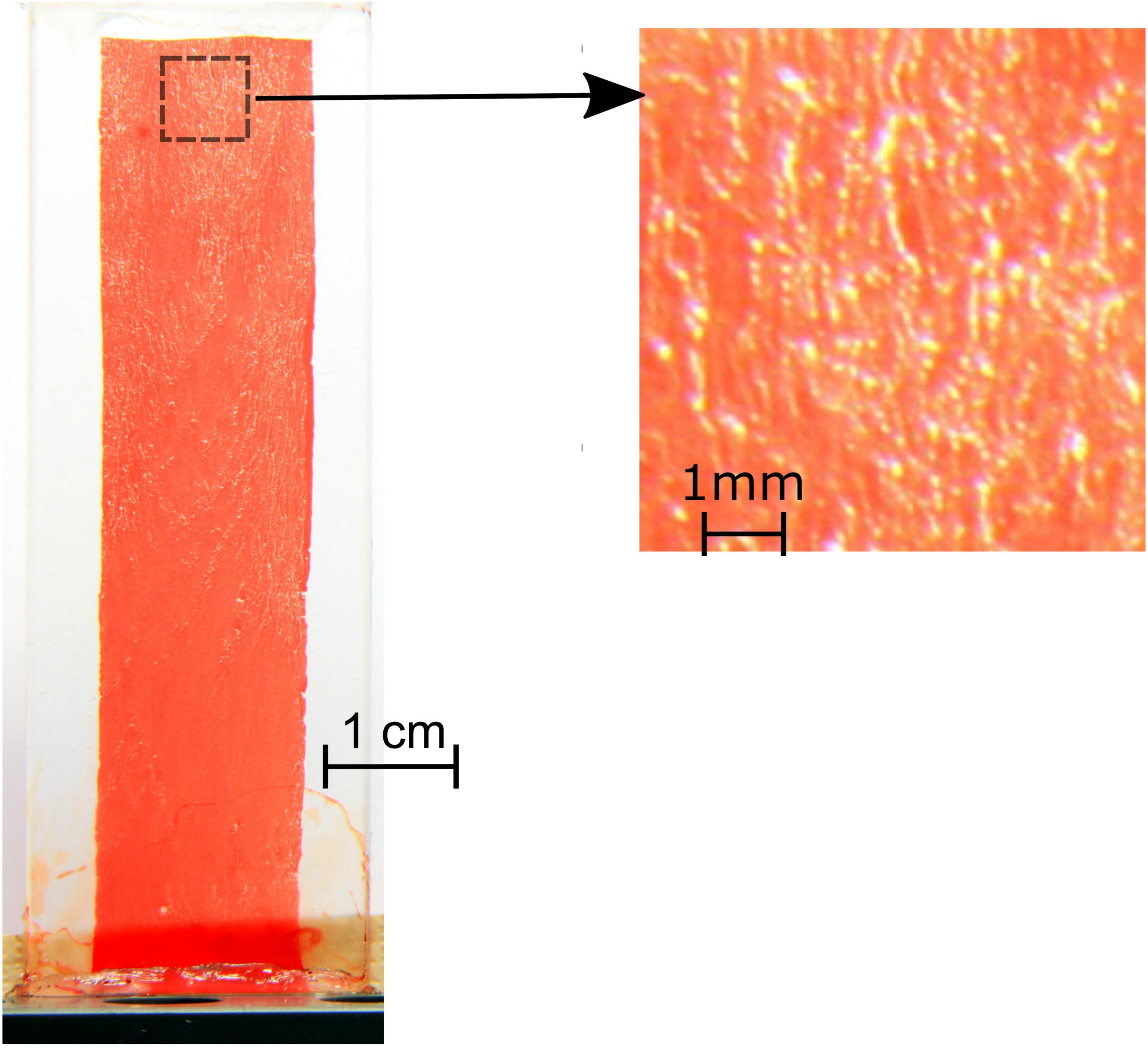

Supplementary Figure S5. Channels that appear in sedimenting blood. The cell glass is approximately 100 µm thick, which makes the cracks visible to the naked eye. The square panel is a magnified view of the squared area in the rectangular picture. These channels are characteristic of the transient gel sedimentation regime of colloidal suspensions.

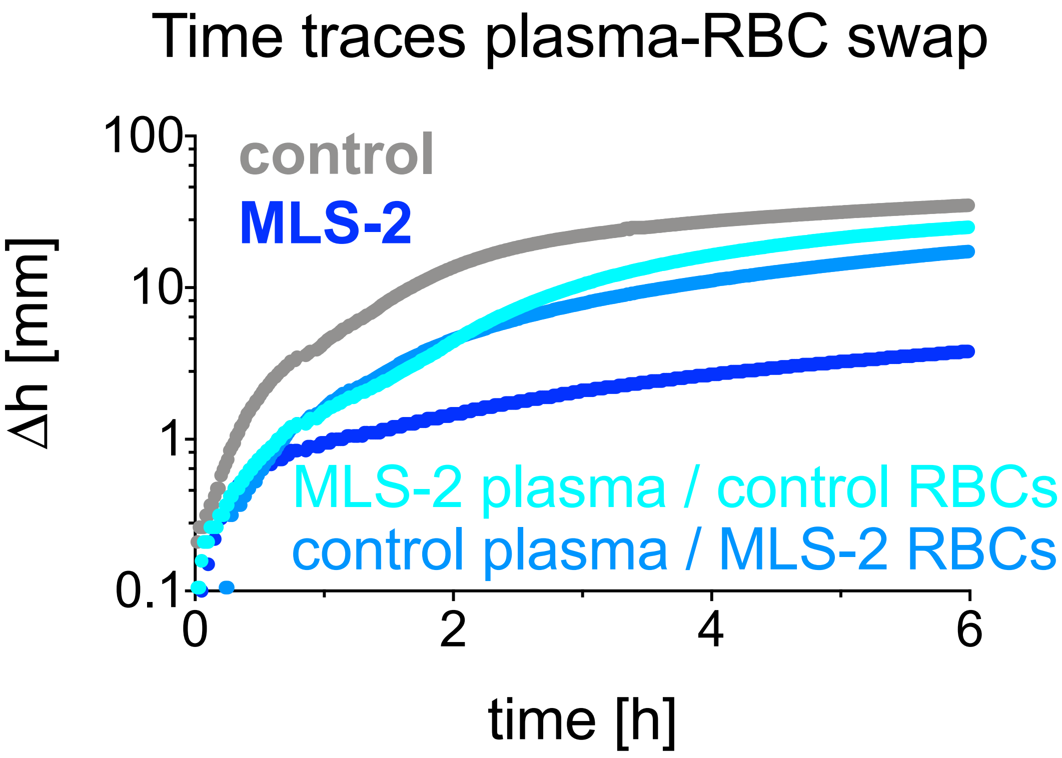

Supplementary Figure S6. Example of time traces obtained by switching control and patient plasma. Erythrocytes from a control subject sediments slower in patient plasma. Erythrocytes from patient MLS-2 patient sediment faster in control plasma.

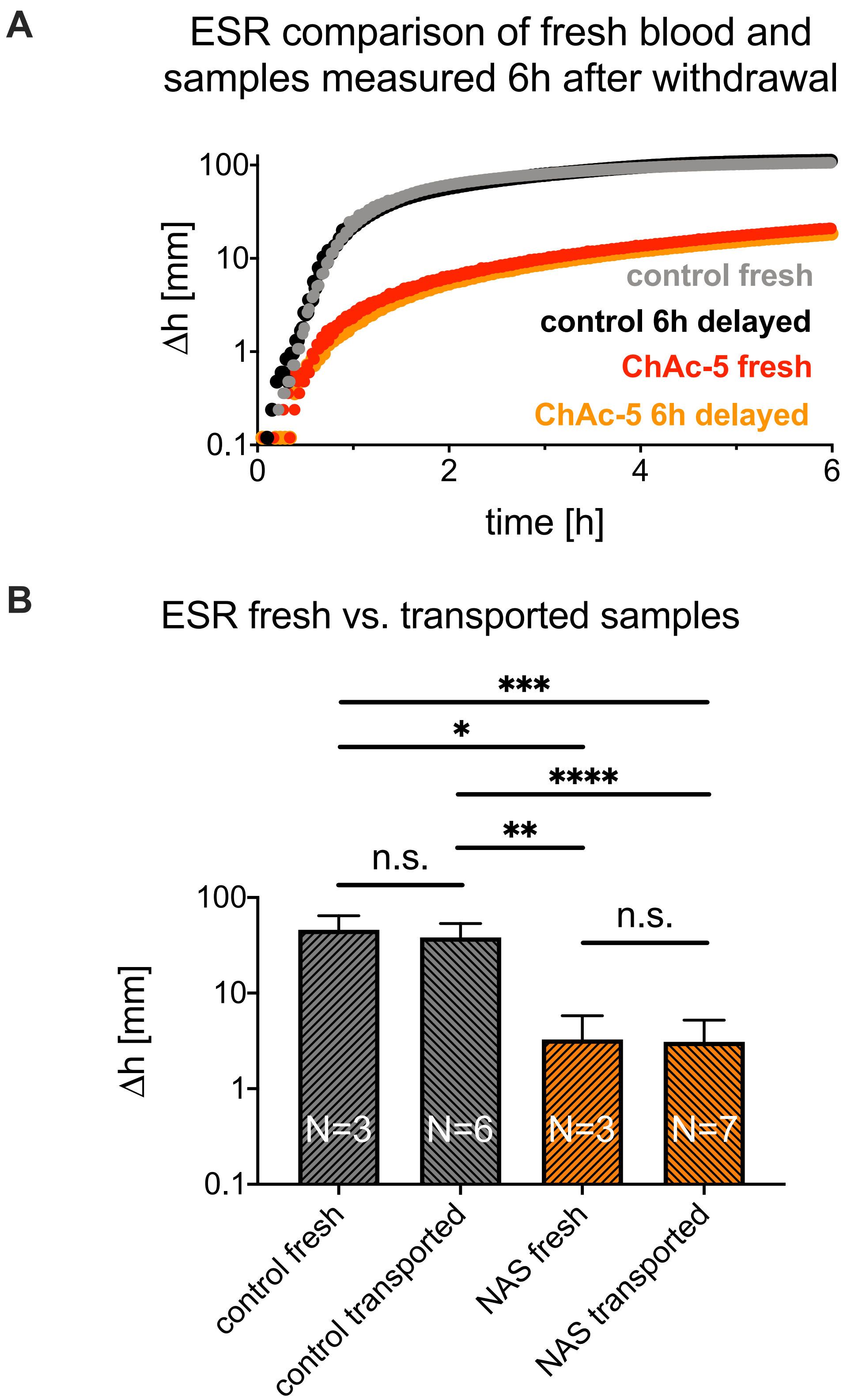

Supplementary Figure S7. Comparison of fresh and delayed observations. A: Comparison of ESR time traces - fresh blood vs. 6 h after withdrawal. Blood samples from one healthy control and patient ChAc-5 were measured immediately after blood withdrawal and 6 h later. The delayed curves are almost indistinguishable from the fresh measurements.B: Comparison of the ESR after 2 h - fresh measurements vs. approximately 6 h of transportation. A subpopulation of the patients traveled to our laboratory in Saarbrücken to allow blood draws for immediate measurement. For the other patients, blood was collected at their local hospitals. We detected no difference in the ESR between fresh and transported blood for either the healthy controls or the NAS patients.

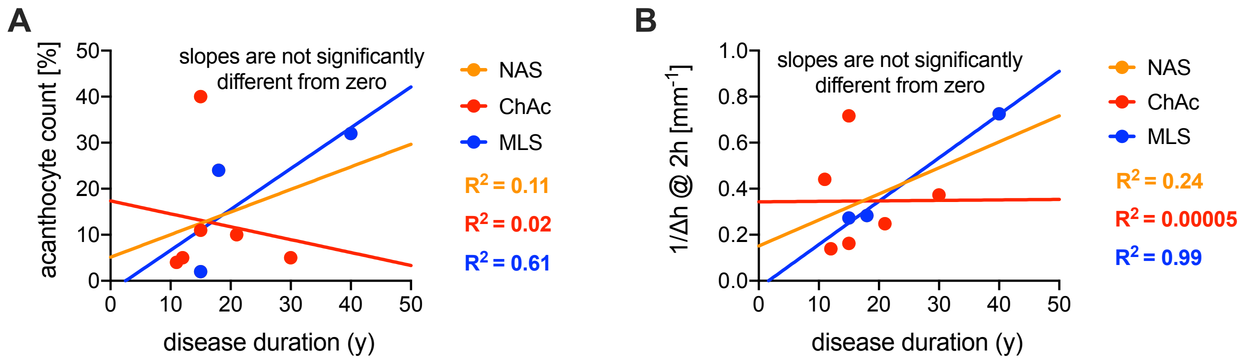

Supplementary Figure S8. Correlation between disease duration and the diagnostic variables. A: Correlation between duration and acanthocytosis number. No significant correlation is observed. B: Correlation between duration and ESR. No significant correlation is observed.
